# Stability of Cerebrospinal Fluid Proteins in Dogs and Cats: Effects of Refrigeration and Freezing Over Time

**DOI:** 10.1111/vcp.70131

**Published:** 2026-07-28

**Authors:** Luana S. Ribeiro, Aude Castel, Bruno B. J. Torres

**Affiliations:** ^1^ Instituto de Ciências Agrárias Universidade Federal dos Vales Do Jequitinhonha e Mucuri Unaí Minas Gerais Brazil; ^2^ Escola de Veterinária e Zootecnia Universidade Federal de Goiás Goiânia Brazil; ^3^ Faculté de Médecine Vétérinaire Université de Montréal Saint‐Hyacinthe Québec Canada

**Keywords:** cats, central nervous system, dogs, maintenance, pyrogallol red method, temperature

## Abstract

**Background:**

The quantification of protein levels is an important part of standard cerebrospinal fluid (CSF) analysis. However, delays in processing due to shipment and/or laboratory opening schedules, combined with the need to preserve sample stability during storage, pose critical challenges to ensuring accurate diagnoses.

**Objectives:**

Our aim was to assess the stability of CSF proteins in canine and feline samples refrigerated and subsequently frozen.

**Methods:**

CSF samples were prospectively collected from dogs and cats. CSF protein levels were measured: immediately after collection; after refrigeration (at 4°C for 48 h); and after freezing (at −20°C for 6 to 150 days). Linear regression was performed for each species to evaluate the influence of time and preservation on protein levels. The impact of the variation in CSF protein measurements on clinical interpretation was evaluated.

**Results:**

Fourteen dogs and four cats with neurological diseases, and five cats euthanized for reasons unrelated to neurological issues were enrolled in the study. Protein measurements were stable over time in dogs (*p* = 0.334) and cats (*p* = 0.785), regardless of the preservation method and timing of analysis. The variation between the CSF protein measurements of fresh and stored samples did not change the interpretation of any results.

**Conclusions:**

CSF protein levels in dogs and cats remained stable when samples were refrigerated for 48 h prior to being frozen for long‐term storage, supporting the feasibility of banking samples for research and laboratory control. Additional studies are necessary to evaluate the stability of specific protein biomarkers.

## 1 Introduction

Protein level is an important parameter in cerebrospinal fluid (CSF) analysis for diagnosing inflammatory, degenerative, or neoplastic processes affecting the central nervous system or nerve roots in dogs and cats [1, 2]. Since many veterinary clinics cannot evaluate CSF in‐house, the sample may need to be sent to a specialized diagnostic laboratory for analysis, resulting in delays in processing [3]. For this reason, ensuring the stability of CSF protein during transport and storage is essential for accurate diagnostics.

Studies in dogs indicated that CSF protein remains relatively stable over time when stored at different temperatures [1, 2, 3]. If a delay in sample processing is expected, it has been recommended that samples be refrigerated at 4°C or frozen at −80°C, allowing analysis within 48 h or 6 months, respectively [2, 3]. However, because baseline CSF protein concentrations are very low, even small analytical fluctuations may occur and can be clinically relevant, particularly when interpreting subtle changes [1]. That being said, the clinical significance of these variations has yet to be thoroughly investigated. Results from these studies have been extrapolated to cats; to the author's knowledge, no research has evaluated the stability of CSF protein in this species. This gap highlights the need for further investigations to establish reliable protocols for feline CSF analysis.

CSF protein level is being investigated as a potential biomarker due to its proximity to the brain, and stored frozen samples can be utilized in research [4, 5]. Although freezing at −80°C has been recommended [2, 3], freezers with this capacity are less commonly available in veterinary clinics and laboratories compared to those at −20°C. Notably, the latter are also used for the preservation of protein biomarkers in dogs, [6] highlighting the need to evaluate the influence of this temperature for the storage of samples intended for subsequent protein analysis.

In this context, the present study aimed to assess the stability of domestic canine and feline CSF proteins in samples refrigerated at 4°C for 48 h and subsequently frozen at −20°C over a period of up to 5 months. We hypothesized that CSF protein levels would remain stable under these conditions without affecting the clinical interpretation of results in both species. With these findings, we hope to establish reliable guidelines for CSF sample handling and analysis, ultimately improving diagnostic accuracy in veterinary practice.

## 2 Materials and Methods

### 2.1 Cerebrospinal Fluid Samples and Collection

CSF samples were prospectively collected from dogs and cats presented to the University Veterinary Hospital of the Faculty of Veterinary Medicine of Montreal University for diagnostic purposes. Additionally, samples were also obtained from the Emergency Service and Intensive Care Unit from animals scheduled for euthanasia to increase the sample size during the study period. For animals undergoing euthanasia, CSF collection was performed immediately after the procedure. Samples were only collected when the procedure could be completed within 30 min after euthanasia. Cases exceeding this timeframe were not included. Samples were only included if sufficient volumes (50 μL) were available from excess to analysis and if the samples could be processed and analyzed immediately after collection. Owner consent was collected in all cases. The study was approved by the Institutional Animal Care and Use Committee of the University (23‐Rech‐2266).

CSF collection was performed at the cerebellomedullary cistern in most cases, except for one dog that collected in the lumbar space. The collections were performed aseptically with a 22G spinal needle, under general anesthesia or after euthanasia, as described in the literature [7].

### 2.2 Protein Measurement

CSF protein levels were measured using the pyrogallol red method on an automatic Chemistry Analyzer UniCel D × C 600 (Beckman Coulter, Brea, California, USA). The detection range was from 0.06 to 3 g/L. Samples exceeding this range were diluted with saline. Samples with high cellularity were centrifuged at 3710 × g for 10 min, and the supernatant was used for measurement.

The analyses were conducted immediately after collection. Subsequently, samples were refrigerated at 4°C for 48 h. A second measurement was performed after this period, and the samples were then frozen at −20°C until all analyses were completed on the same day, within a timeframe of 6 to 150 days. CSF protein level was considered increased when the values were higher than 0.30 g/dL for cerebellomedullary punctions and higher than 0.45 g/dL for CSF collected at the lumbar space [6].

### 2.3 Statistical Analysis

Descriptive statistics were performed for qualitative data (signalment and time of CSF protein measurement of frozen samples). To assess the influence of time on protein levels, linear regression analyses were conducted for each species. A significant level of 0.05 was established. Statistical analysis was performed using R software (version 4.3.3, R Core Team, 2019). Additionally, the impact of variations in CSF protein measurements between fresh and stored samples on clinical interpretation was evaluated.

## 3 Results

Twenty‐three animals met the inclusion criteria and were enrolled in the study, including 14 dogs and nine cats. All the dogs and four cats had neurological signs, while the remaining cats were euthanized due to various terminal conditions unrelated to neurological issues. Three CSF samples from dogs were not measured for protein 48 h after refrigeration because they were obtained from a previous study conducted by our research team. In addition, three CSF samples from cats were not evaluated after being frozen due to insufficient sample volume (Tables 1 and 2).

**TABLE 1:** Identification and results of CSF protein levels in dogs, measured immediately after collection, after 48 h of refrigeration, and at various points post‐freezing. The duration of freezing in days is indicated for each sample.

ID	Breed	Gender and Neuter status	Age (months)	BW (kg)	Diagnosis or suspicion	CSF proteins levels (mg/dL)			Duration of freezing (days)
						Immediately after collection	Refrigerated at 4°C for 48 h	Frozen at −20°C	
D1	Golden retriever	Female spayed	127	10.1	Undefined	14	NA	15	150
D2	Golden retriever	Female spayed	24	23.2	Neuronal ceroid lipofuscinosis^1^	18	NA	13	144
D3	Pit bull	Male neutered	102	29.7	Degenerative radiculomyelopathy^1^	15	15	12	125
D4	Mixed breed	Female spayed	21	41.2	Undefined	12	12	12	118
D5	Labrador retriever	Male intact	128	37.4	Degenerative radiculomyelopathy^1^	26	24	23	113
D6	French bulldog	Male intact	36	NA	Acute extrusion of the nucleus pulposus^3^	150	NA	145	61
D7	German shepherd	Female spayed	169	33	Neoplasm^3^	38	38	42	52
D8*	Pit bull	Female intact	16	23.4	Steroid responsive meningitis‐arteritis^2^	70	69	87	45
D9	Siberian husky	Male neutered	116	30.9	Idiopathic epilepsy^1^	18	17	25	44
D10	Bernese mountain dog	Female spayed	48	43	Choroid plexus tumor associated with hydrocephalus^1^	749	741	741	33
D11	Chihuahua (mixed breed)	Male neutered	180	NA	Undefined	106	105	127	27
D12	Yorkshire terrier	Male neutered	103	3.5	Idiopathic epilepsy^2^	8	7	7	13
D13	Boston terrier (mixed breed)	Female spayed	84	24.5	Oligodendroglioma^1^	306	316	310	25
D14	Australian shepherd	Male neutered	18	22	Disseminated Blastomycosis^1^	61	60	62	6

Abbreviations: *, lumbosacral space cerebrospinal fluid punction; 1, histopathological diagnosis; 2, clinical diagnosis; 3, clinical suspicion; NA, information not available.

**TABLE 2:** Identification and results of CSF protein levels in cats, measured immediately after collection, after 48 h of refrigeration, and at various points post‐freezing. The duration of freezing in days is indicated for each sample.

ID	Breed	Gender	Neuter status	Age (months)	BW (kg)	Diagnosis or suspicion	CSF proteins levels (mg/dL)			Duration of freezing (days)
							Immediately after collection	Refrigerated at 4°C for 48 h	Frozen at −20°C	
C1	Domestic Shorthair	NA	NA	24	6.2	Spinal cord trauma^3^	48	45	39	133
C2	Domestic Shorthair	Female	Spayed	109	4.38	Neoplasm^2^	11	3	NA	NA
C3	Domestic Shorthair	Male	Neutered	60	NA	UN	13	12	12	112
C4	Domestic Shorthair	Male	Neutered	204	NA	UN	15	14	NA	NA
C5	Domestic Shorthair	Female	Spayed	218	2.88	UN	26	27	25	84
C6	Domestic Shorthair	Male	Neutered	168	7.1	UN	32	32	31	32
C7	Domestic Shorthair	Female	Spayed	180	NA	Undefined	33	32	35	26
C8	Persian cat	Male	Neutered	192	NA	UN	102	104	98	25
C9	Domestic Shorthair	Male	Neutered	144	NA	Undefined	23	24	NA	NA

Abbreviations: 1, histopathological diagnosis; 2, clinical diagnosis; 3, clinical suspicion; NA, information not available; UN, euthanized due to various terminal conditions unrelated to neurological issues.

CSF protein concentration at each time and mean duration of freezing are presented in Table 3. Protein measurements remained stable over time for both dogs (*p* = 0.334) and cats (*p* = 0.785), regardless of the timing of analysis (Figure 1). The average variation in results between fresh and refrigerated samples was 2 mg/dL (range: 0 to 1 mg/dL) for dogs and 2 mg/dL (range: 0 to 8 mg/dL) for cats. In frozen samples, the average variation was 6 mg/dL (range: 0 to 21 g/dL) for dogs and 3 mg/dL (range: 1 to 9 mg/dL) for cats. Variations observed in protein concentrations sometimes increased and sometimes decreased over time, with fluctuations observed in both refrigerated and frozen conditions. However, the variation found between the CSF protein measurements in fresh and stored samples did not change the interpretation of any results.

**TABLE 3:** Median of CSF protein levels in dogs and cats, measured immediately after collection, at 48 h following refrigeration, and at different times after freezing. The table also includes the median and range of the number of days between sample collection and CSF protein measurement after freezing for the cat and dog groups.

Animal	Cerebrospinal protein (mg/dL)			Time of CSF protein mensuration of frozen samples (day)
	Immediately after collection	48 h after refrigeration	After freezing	
Canine	3 (8–749)	4 (7–741)	3 (7–741)	48.5 (6–150)
Feline	3 (11–102)	3 (3–104)	3 (12–98)	58 (25–133)

**FIGURE 1:**
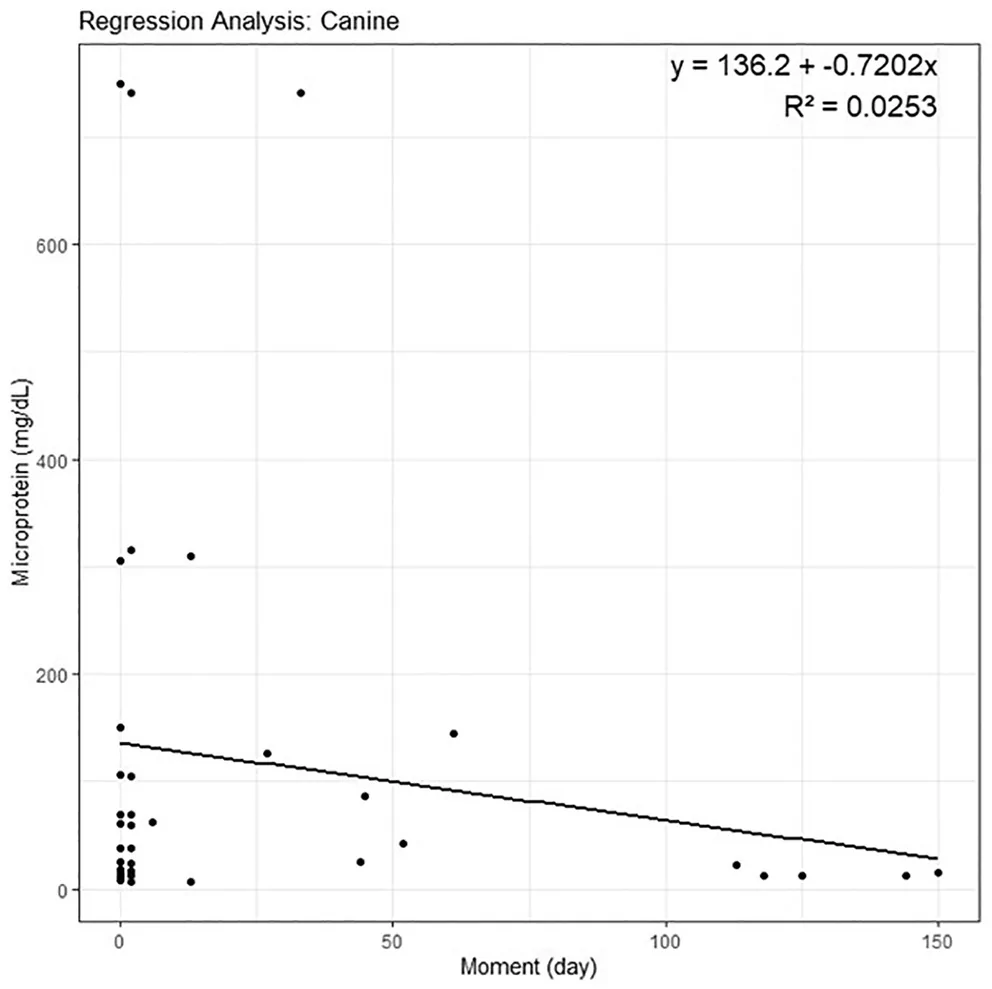
Regression plot of CSF protein levels in dogs, measured immediately after collection, after 48 h of refrigeration, and at various points post‐freezing. The results demonstrate that protein levels remained stable over time, regardless of the measurement moment.

Increased protein levels were found in seven dogs (7/14, 50%, 38–749 mg/dL) and four cats (4/9, 44%, 32–102 mg/dL). Of these, six dogs and one cat had significantly increased protein (defined as protein levels above twice the reference value). Among those dogs, the average variation between fresh and refrigerated samples was 5 mg/dL (range: 1 to 10 mg/dL), and between fresh and frozen samples was 7.8 mg/dL (range: 1 to 10 mg/dL). For the cat, the difference was 2 mg/dL for refrigerated and 4 mg/dL for frozen samples, Figure 2 compared with fresh samples.

**FIGURE 2:**
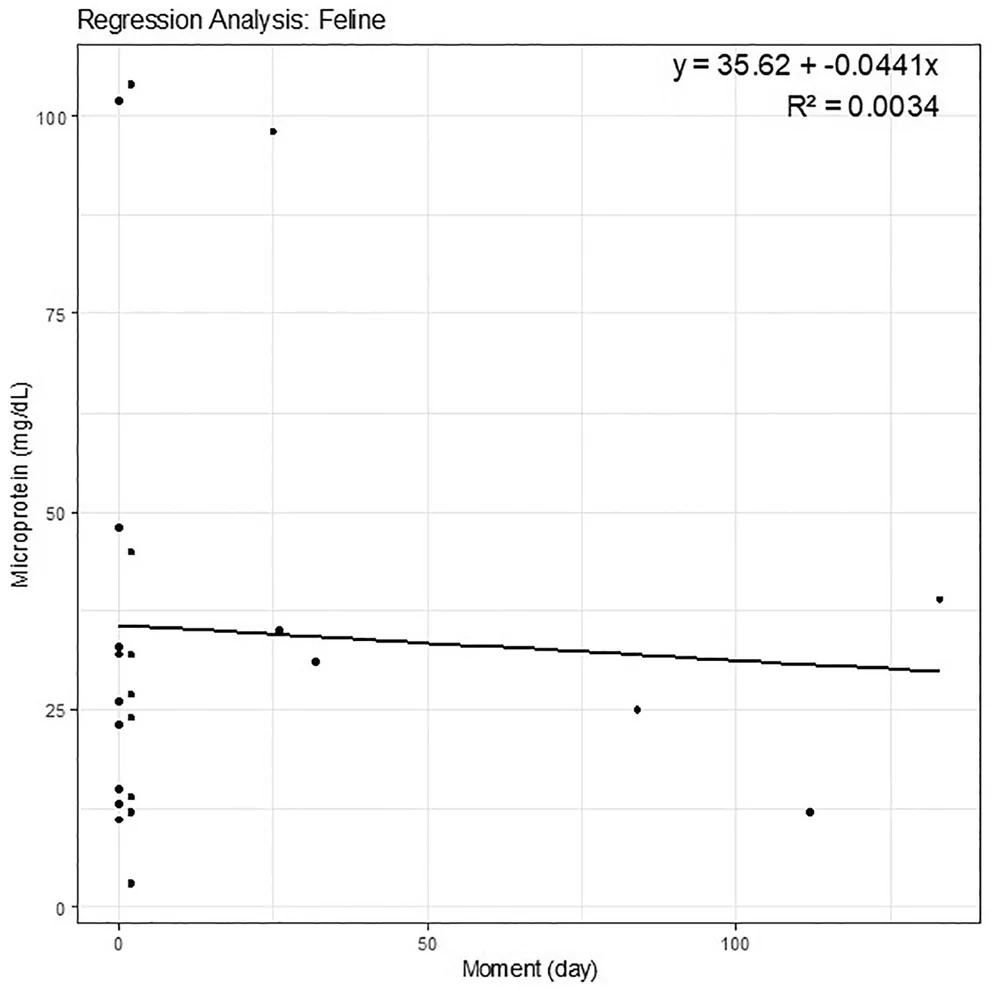
Regression plot of CSF protein levels in cats, measured immediately after collection, after 48 h of refrigeration, and at various points post‐freezing. The results demonstrate that protein levels remained stable over time, regardless of the measurement moment.

## 4 Discussion

CSF protein levels remained stable after refrigeration at 4°C for 48 h and subsequent freezing at −20°C over time in both dogs and cats. Similar findings were observed in healthy and neurologically affected dogs with refrigerated samples [1, 3]. In frozen samples, one study reported that normal protein measurements in dogs remained stable for up to 1 month at −4°C [1]. Another study evaluated CSF protein in dogs affected by neurological diseases and found that it remains stable for up to 6 months at −80°C [2]. To the best of our knowledge, this is the first study evaluating CSF protein stability in freezers at −20°C in dogs. In cats, this is the first study to evaluate CSF protein stability over time in both refrigeration and freezing (−4°C) conditions.

Determining total protein concentration is a crucial aspect of canine CSF analysis, as it can reflect inflammatory, degenerative, or neoplastic processes [1]. Given that normal CSF has a low protein concentration, even subtle alterations in this parameter can carry significant clinical implications. A study examining CSF samples from dogs found that, while no statistically significant differences in mean CSF protein levels were observed after storage at different temperatures (25°C, 4°C, and −4°C) for up to 1 month, there was considerable variability in these means [1]. However, since the study evaluated CSF protein levels in healthy dogs, the clinical relevance of this variability was not assessed. In our current study, we found that differences in CSF protein levels between fresh and stored samples did not demonstrate clinically significant importance for both normal and increased protein samples from dogs and cats over a period of up to 5 months. Furthermore, the small fluctuations observed were within the estimated error of the assay itself.

Higher protein concentrations are commonly associated with pleocytosis, and identifying the predominant cell type helps guide the differential diagnosis [7]. In the present study, most dogs showed increased protein levels accompanied by pleocytosis, with two cases (D11 and C1) also presenting blood contamination. However, elevated protein can also occur in the absence of pleocytosis, a phenomenon known as albuminocytological dissociation [8]. Although this finding may support the presence of disease, albuminocytological dissociation alone has limited value in determining the underlying etiology. This alteration was observed in only three dogs (D7, D13, and D14), all of which were diagnosed with neoplasia, a condition reported as a common cause of albuminocytological dissociation in dogs [9]. Notably, the highest CSF protein concentrations in dogs were also associated with neoplastic processes. Nevertheless, these findings should be interpreted with caution, as neoplasia was the most frequent diagnosis in dogs in this study.

It is recommended that CSF analysis be conducted within 30 to 60 min of collection. However, some veterinary centers must send samples to specialized diagnostic laboratories, resulting in potential delays of 4 to 8 h and even longer during non‐business hours [3]. Additionally, a recent study in dogs has demonstrated that CSF proteins should not be evaluated using urinary dipsticks, highlighting the limitations of in‐house testing methods [10]. In veterinary practice, CSF samples are refrigerated until they can be analyzed for protein [3]. Typically, this delay can extend to 48 h (e.g., over weekends), which is the time frame often encountered in routine clinical settings. During this period, in our study, CSF samples showed stability in both dogs and cats. Consequently, CSF samples may encounter delays in frozen storage [4]. Notably, CSF protein measurements did not exhibit significant variation in frozen samples at −20°C after a 48°h delay compared to fresh or refrigerated, remaining stable for up to 150 days in dogs and 133 days in cats, which demonstrates the viability of batching these samples for research or laboratory control. It is important to emphasize that studies on the stability of specific protein biomarkers should be conducted, like those performed in human medicine [5].

The most significant limitation of this study was the inclusion of a low number of cases, particularly for cats. Despite the small sample size, there was good stability of refrigerated and frozen CSF proteins in both species, with only minor differences in measurements that did not affect the interpretation of the results. Additionally, both species yielded representative outcomes, with approximately half of the patients exhibiting normal results and half showing increased protein. A post hoc power analysis based on the observed effect sizes (*f* ^2^ = 0.0259 for canines and *f* ^2^ = 0.0035 for felines) indicated that approximately 303 canine and 2270 feline cases would be required to achieve 80% statistical power at an alpha level of 0.05. Given the small effect sizes observed, reaching such sample sizes would be unrealistic in routine clinical settings. Thus, the low power of the current study reflects the minor impact of storage time on CSF protein concentrations, rather than a failure to detect clinically relevant differences.

Another limitation is the lack of standardization regarding the freezing duration of the samples. However, most samples were stored for an extended period, with 70% (14 out of 20) stored for more than 1 month and 15% (3 out of 20) stored between 25 and 27 days. The remaining samples included one stored for 6 days and two stored for 13 days. We could also mention as a minor limitation that most CSF samples were collected from the cerebellomedullary cistern and only one from the lumbar space, although this factor is not expected to interfere with the stability of CSF protein concentrations.

In conclusion, this study shows that CSF protein levels in dogs and cats can remain reliable when refrigerated at 4°C for up to 48 h and after long‐term freezing at −20°C. While previous research has confirmed protein stability in dogs, this is the first study to examine this in cats, addressing an important gap in veterinary knowledge. Notably, we found that CSF protein stability was maintained even after a 48°h period before freezing, suggesting the feasibility of banking samples for research and laboratory control. However, additional studies are necessary to evaluate the stability of specific protein biomarkers.

## Funding

This work was supported by Coordenação de Aperfeiçoamento de Pessoal de Nível Superior and PremieRpet Institute.

## Conflicts of Interest

The authors declare no conflicts of interest.

## Data Availability

The data that support the findings of this study are available on request from the corresponding author. The data are not publicly available due to privacy or ethical restrictions.
